# Response to Khodamoradi: Methodological clarifications regarding NanOx predictions in helium SOBP

**DOI:** 10.1016/j.phro.2026.101088

**Published:** 2026-09-21

**Authors:** Thomas Berger, Gersende Alphonse, Mario Alcocer-Avila, Caterina Monini, Charbel Koumeir, Claire Rodriguez-Lafrasse, Nicolas Varmenot, Vincent Métivier, Ferid Haddad, Jean Michel Létang, Étienne Testa

**Affiliations:** a Universite Claude Bernard Lyon 1, CNRS/IN2P3, IP2I Lyon, UMR 5822, F-69100 Villeurbanne, France; b Radiation Oncology Department, Center Hospitalier Lyon Sud, Hospices Civils de Lyon, Pierre Bénite, France; c Department of Biochemistry and Molecular Biology, Lyon-Sud Hospital, Hospices Civils de Lyon, Pierre-Bénite, France; d Laboratoire SUBATECH, UMR 6457 CNRS-IN2P3, IMT Atlantique, Nantes Université, France; e GIP ARRONAX, Saint Herblain, France; f INSERM, U1232, CRCI^2^NA, Nantes, France; g Institut de Cancérologie de l'Ouest, F-44800 Saint-Herblain, France.; h INSA-Lyon, Université Claude Bernard Lyon 1, CREATIS, CNRS UMR5220, Inserm U1294, 69373 Lyon, France.

We thank Khodamoradi for sharing their understanding of our methods and for expressing their concerns regarding the validity of our conclusions [1]. However, as we clarify below, both criticisms are based, at least in part, on misunderstandings of our methodology.

Khodamoradi's first concern relates to the use of SQ20B data for tuning the NanOx model and the subsequent use of the tuned model to predict survival in the same cell line. However, characterizing this as ‘circular validation’ or a ‘mere interpolation exercise’ overlooks the fact that surviving fraction depends not only on the cell line, but also on the characteristics of the irradiation beam, including ion species and LET distribution. Importantly, and as specified in our article [2], the model was tuned using experimental data from proton and carbon-ion irradiation, whereas our study assessed its predictions for helium-ion irradiation. The model was therefore tested against helium-ion SQ20B survival data that were not used for its parameterization. This therefore goes beyond simply reproducing or interpolating the data used for model parameterization and provides a genuine challenge to the model.

More broadly, prior use of a cell line or ion species in model development does not, in itself, negate the scientific value of an independent experiment using the same cell line and ion species. To go one step further, even exact replication can provide information on the reproducibility of experimental findings.

The second concern raised by Khodamoradi results from a misunderstanding of how dose was determined in our study. The HDV2 radiochromic films were not used to quantify the absolute dose delivered to the cells, but, as stated in our article [2], to characterize the helium beam quality, specifically the depth and lateral homogeneity of the SOBP. Absolute dose was instead determined independently using an indirect dosimetry method. Briefly, the number of K-shell X-rays emitted from a copper foil by the helium beam was measured with a silicon drift detector and related to the number of incident helium ions. The corresponding dose calibration was established using a Faraday cup positioned in place of the cell well. Thus, while the potential LET-dependent response of the radiochromic films may have affected the accuracy of the beam characterization, as acknowledged in our article, it does not affect the absolute dose determination used for the survival measurements.

Khodamoradi also repeatedly characterizes our study as an attempt to validate the NanOx model. However, throughout the manuscript, we avoided such unqualified terminology, reported the model-experiment differences quantitatively, and discussed relevant sources of uncertainty rather than presenting our findings as definitive validation of NanOx. Our conclusion likewise states that “these results support further validation of the model in a broader range of cell lines and helium ion irradiation configurations,” explicitly acknowledging the need for additional studies to assess its broader applicability.

We welcome critical discussion of our work. Nevertheless, we believe that clarification of these points at the outset could have provided important context and avoided the strong conclusions drawn from these misunderstandings.

## Declaration of generative AI in the manuscript preparation process

During the preparation of this work, the authors used ChatGPT v5 to improve the grammar, readability, flow, and clarity of the manuscript. After using this tool, the authors reviewed and edited the content as needed and take full responsibility for the content of the published article.

## CRediT authorship contribution statement

**Thomas Berger:** Writing – original draft. **Gersende Alphonse:** Writing – review & editing. **Mario Alcocer-Avila:** Writing – review & editing. **Caterina Monini:** Writing – review & editing. **Charbel Koumeir:** Writing – review & editing. **Claire Rodriguez-Lafrasse:** Writing – review & editing. **Nicolas Varmenot:** Writing – review & editing. **Vincent Métivier:** Writing – review & editing. **Ferid Haddad:** Writing – review & editing. **Jean Michel Létang:** Writing – review & editing. **Étienne Testa:** Writing – review & editing.

## Declaration of competing interest

The authors declare that they have no known competing financial interests or personal relationships that could have appeared to influence the work reported in this paper.
